# Antifungal and Zearalenone Inhibitory Activity of *Pediococcus pentosaceus* Isolated from Dairy Products on *Fusarium graminearum*

**DOI:** 10.3389/fmicb.2016.00890

**Published:** 2016-06-13

**Authors:** Muthulakshmi Sellamani, Naveen K. Kalagatur, Chandranayaka Siddaiah, Venkataramana Mudili, Kadirvelu Krishna, Gopalan Natarajan, Venkata L. Rao Putcha

**Affiliations:** ^1^Toxicology and Immunology Division, DRDO-BU-Centre for Life sciences, Bharathiar University Campus, CoimbatoreIndia; ^2^Food Microbiology Division, Defence Food Research LaboratoryMysore, India; ^3^Department of Biotechnology, University of MysoreMysore, India; ^4^Defence Bioengineering and Electromedical LaboratoryBangalore, India; ^5^Food Biotechnology Division, Defence Food Research LaboratoryMysore, India

**Keywords:** *F. graminearum*, Zearalenone, *P. pentosaceus*, minimum inhibitory concentration, dual culture overlay technique, ergosterol, reactive oxygen species, reverse transcriptase qPCR analysis

## Abstract

The present study was aimed to evaluate the bio-control efficacy of *Pediococcus pentosaceus* isolated from traditional fermented dairy products originated from India, against the growth and zearalenone (ZEA) production of *Fusarium graminearum*. The cell-free supernatants of *P. pentosaceus* (PPCS) were prepared and chemical profiling was carried out by GC-MS and MALDI-TOF analysis. Chemical profiling of PPCS evidenced that, the presence of phenolic antioxidants, which are responsible for the antifungal activity. Another hand, MALDI-TOF analysis also indicated the presence of antimicrobial peptides. To know the antioxidant potential of PPCS, DPPH free radical scavenging assay was carried out and IC_50_ value was determined as 32 ± 1.89 μL/mL. The antifungal activity of *P. pentosaceus* was determined by dual culture overlay technique and zone of inhibition was recorded as 47 ± 2.81%, and antifungal activity of PPCS on *F. graminearum* was determined by micro-well dilution and scanning electron microscopic techniques. The minimum inhibitory concentration (MIC) of PPCS was determined as 66 ± 2.18 μL/mL in the present study. Also a clear variation in the micromorphology of mycelia treated with MIC value of PPCS compared to untreated control was documented. Further, the mechanism of growth inhibition was revealed by ergosterol analysis and determination of reactive oxygen species (ROS) in PPCS treated samples. The effects of PPCS on mycelial biomass and ZEA production were observed in a dose-dependent manner. The mechanism behind the suppression of ZEA production was studied by reverse transcriptase qPCR analysis of ZEA metabolic pathway genes (*PKS4* and *PKS13*), and results showed that there is a dose dependent down-regulation of target gene expression in PPCS treated samples. The results of the present study were collectively proved that, the antifungal and ZEA inhibitory activity of PPCS against *F. graminearum* and it may find a potential application in agriculture and food industry as a natural bio-controlling agent.

## Introduction

Mycotoxins are toxic secondary metabolites of filamentous fungi and predominantly produced by *Aspergillus*, *Penicillium*, and *Fusarium* species (Venkataramana et al., 2013, 2014; Priyanka et al., 2014). The fungal infestation of agricultural commodities usually occurs during pre and post-harvesting sessions as a result of improper handling, transportation and storage practices (Morgavi et al., 2007). The Food and Agriculture Organization (FAO) of the United Nations reported that about 25% of agricultural products throughout worldwide were contaminated with fungi and mycotoxins (McKellar and Lu, 2003). The fungal infestation of agricultural commodities could result in the loss of grain texture, color, nutritional quality, and finally brings immense financial loss to the farming society (Morgavi et al., 2007). Consumption of mycotoxins contaminated food and feed could cause acute and chronic mycotoxicosis, including teratogenic, carcinogenic, oestrogenic, neurotoxic, and immunosuppressive effects (Morgavi et al., 2007; Venkataramana et al., 2015). Due to the deleterious effects of toxigenic fungi and their toxins present in the food grains and food products, it could be considered as a great threat to the society in terms of microbiological safety and food security (Pitt and Hocking, 2009).

Among the toxigenic fungal species, *Fusarium graminearum* was most considerable due to the high prevalence of different agro-climatic conditions with the ability to produce multiple mycotoxins, including zearalenone (ZEA), deoxynivalenol (DON), and nivalenol (NIV) (Morgavi et al., 2007; Ramana et al., 2011; Bernhoft et al., 2012; Venkataramana et al., 2014). The ZEA also known as an F-2 toxin and only reported non-steroidal estrogenic mycotoxin produced by *F. graminearum* in temperate climatic conditions (Zinedine et al., 2007). ZEA binds strongly with 17β-estradiol of cytosolic estrogen receptors present in the uterus and mammary gland and causes reproductive disorders such as the expansion of ovaries and uterus, vulvovaginitis and immature births in farm animals, and also occasionally responsible for hyperoestrogenism in humans (Minervini and Dell’Aquila, 2008). Recent reports on ZEA toxicity revealed that it could cause genotoxic (Gao et al., 2013), hepatotoxic (Stadnik and Borzecki, 2009), immunotoxic (Salah-Abbès et al., 2010), and neurotoxic effects (Venkataramana et al., 2014) in humans and animals. The international agency for research on cancer (IARC) also reported the carcinogenic properties of ZEA in laboratory animals and categorized as a Group 3 carcinogen (IARC, 1999). Keeping these toxicological aspects of ZEA, regulatory agencies throughout the worldwide proposed the maximum permissible limits in food and feed matrices. The joint FAO/WHO Expert Committee on Food Additives (JECFA) has suggested a Provisional Maximum Tolerable Daily Intake (PMTDI) of 0.5 μg/kg body weight (JECFA, 2000). The European Union (EU) has recognized permissible limits for ZEA in unprocessed cereals as 100 μg/kg excluding maize and in unprocessed maize was 350 μg/kg (European Commission, 2007). However, reports of Pleadin et al. (2012) revealed that maize grains were contaminated with high levels of 5.11 mg/kg ZEA and also Yoshinari et al. (2014) reported that Job’s tears products of cereals have been found as 3.1 to 5.9 μg/kg of ZEA. These reports, further warns that there is an immediate need to develop a cost-effective bio-control strategy for successive mitigation of *F. graminearum* and ZEA contamination in food grains intended for consumption.

Currently, the use of synthetic antifungal agents for control of fungi is in practice. However, due to consumer safety and awareness, the use of chemical and synthetic fungicides is a great threat to the society due to increasing in multiple drug resistant microbes, environmental pollution and its residual toxicity to animals and humans (Kretschmer et al., 2009). Therefore, there is a trend to use bio-agents to control the fungal growth and the elimination of mycotoxins in food and feed commodities by several agricultural and food industries (Raut and Karuppayil, 2014). In this connection, several researchers were reported the use of microorganisms such as yeast, filamentous fungi and bacteria as antifungal agents (Dalie et al., 2010), and inhibitors of mycotoxin synthesis (Gourama and Bullerman, 1995; Gourama, 1997) as well as mycotoxin detoxifying agents (Fuchs et al., 2008; Wang et al., 2015). With this instance, Lactic acid bacteria (LAB) has been used as a safe bio-preservative agent for storing several food products through globally. LAB was declared as GRAS (generally recognized as safe) by FDA (Food and Drug Administration) and also as QPS (qualified presumption of safety) status by EFSA (European Food Safety Authority). LAB plays a major role in the preservation of fermented food products by maintaining the microbial stability through their antimicrobial active metabolites such as organic acids (mainly lactic acid and acetic acid), reuterin, phenyllactic acid, fatty acids, and bacteriocins (Dalie et al., 2010). LAB also exhibited potential features such as antifungal, inhibitors of mycotoxin synthesis, and mycotoxin detoxifying agents, which are much required in agricultural and food sector. Gourama and Bullerman (1995) reported the antifungal and anti-aflatoxin production properties of lactic acid and other active metabolites of LAB. Lavermicocca et al. (2000) also reported the antifungal activity of phenyllactic acid and 4-hydroxyl-phenyl-lactic acid isolated from *Lactobacillus plantarum.* Similarly, Dalie et al. (2010), observed the inhibition of growth and fumonisins production of *F. proliferatum* and *F. verticillioides* by *P. pentosaceus* (L006) isolated from maize leaf. Recently Wang et al. (2015) reported that LAB strains possess the detoxification property of mycotoxins through binding mechanism to the bacterial cell wall components such as polysaccharides and proteins. In addition to this, Peltonen et al. (2001) reported the binding properties of dairy strains toward aflatoxin B1 and also Fuchs et al. (2008) reported the detoxification of patulin and ochratoxin A in aqueous solution by *L. acidophilus* VM 20 and *B. animalis* VM 12.

Owing to the above, the present study was aimed to evaluate the inhibitory activity of *P. pentosaceus* culture supernatant (PPCS) on the growth and ZEA production of *F. graminearum*. The chemical profiling of PPCS was carried out by MALDI-TOF and GC-MS methods. Antioxidant activity of PPCS was determined by DPPH free radical scavenging assay and antifungal activity was determined by micro-well dilution, well-diffusion and scanning electron microscopic (SEM) methods. The mechanism of growth inhibitory activity of PPCS on *F. graminearum* was revealed by determining the ergosterol content and reactive oxygen species (ROS) production. The ZEA synthesis inhibitory activity of PPCS at genetic level was determined by the reverse transcriptase qPCR (Rt-qPCR) method as well as UHPLC analysis of ZEA.

## Materials and Methods

### Chemicals and Media

Potato dextrose (PD) agar and broth, Czapek–Dox (CD) agar and broth, de Man, Rogosa and Sharpe (MRS) agar and broth, malt extract soft (MES) agar, peptone and 2,2-diphenyl-1-picrylhydrazyl (DPPH) were obtained from HiMedia, (Mumbai, India). Dichloro-dihydro-fluorescein diacetate (DCFH-DA), α-cyano-4-hydroxy cinnamic acid, trifluoroacetic acid (TFA), methanol, ergosterol and zearalenone (ZEA) were obtained from Sigma–Aldrich (Bangalore, India). All the plasticware used in the study were acquired from Eppendorf (Bangalore, India), and other chemicals and solvents used in the study were fine grade and acquired from Merck Millipore (Bangalore, India).

### Screening and Isolation of Lactic Acid Bacteria

The dairy products including paneer, khoa, peda, kulfi, ghee, kheer, and yogurt were collected from local markets of Coimbatore (Tamil Nadu, India) and used for isolation LAB. A 10 mg of samples were homogenized with 9 mL of sterile distilled water containing 0.85% NaCl and 0.1% peptone. A 10-fold serial dilutions were made from 10^-1^ to 10^-7^ and spread plated on MRS agar and incubated at 37°C for 48 h under aerobic condition. The colonies were picked randomly and streaked plated on MRS agar and incubated for 48 hrs at 37°C. The pure colonies were grown in MRS broth and stored at 4°C for further studies.

### Molecular Identification of LAB

The genomic DNA was extracted from the overnight grown culture of LAB using genomic DNA extraction kit (Roche life science, USA) following the instructions of the manufacturer. The PCR was set for extracted DNA using 16S rRNA universal primers (**Table 1**) and briefly, 20 μL volume of reaction mixture included: 2 μL of PCR reaction buffer (10X), 0.2 μL of Taq polymerase (1 unit/1 μL), 1 μL of 16S rRNA primers (500 nM), 1 μL of template DNA (100 ng) and 15.8 μL of nuclease-free water (PCR grade). The PCR reaction was carried out in Mastercycler nexus (Eppendorf, Germany) with following conditions: initial denaturation at 94°C for 3 min, denaturation at 94°C (45 s), annealing at 55°C (1 min), elongation at 72°C for 2 min and final extension at 72°C for 10 min up to 35 cycles. The PCR product was purified using High Pure PCR Product Purification Kit following the instructions of the manufacturer (Roche life science, USA) and sequenced at Eurofins Genomics India Pvt Ltd (Bangalore, India). The molecular identification of LAB strains was determined using BLAST analysis.

**Table 1:** Primer sequences were used in the present study.

Target Gene	Primer sequence (5′ to 3′)	Tm (°C)
*GAPDH*-F	TATCACGTCTGCCACGAT	56
*GAPDH*-R	CATGTAGGCCTGTGATGA	
*PKS13*-F	TTACCCGCCTCGTTAAAG	56
*PKS1*3-R	AGCTGGCTAAGCGAGGCA	
*PKS4*-F	ATCGGTCATCTTGAGGCT	58
*PKS4*-R	CCGTAGAGAATGCTTTGT	
27F	AGAGTTTGATCM (A/C) TGGCTCAG	56
1492R	TACCTTGTTACGACTT	

### Characterization of *P. pentosaceus* Culture Supernatant (PPCS)

*Pediococcus pentosaceus* was grown in 100 mL of MRS broth in 250 mL Erlenmeyer flask at 37°C for 48 h under aerobic condition. The cell-free supernatant was collected by centrifugation (8000 rpm for 10 min at 4°C) and filtered through a sterile filter (0.22 μm, Millipore) and referred as PPCS. The PPCS was concentrated by ammonium sulfate precipitation method and the precipitate was dialyzed against 0.5 mM phosphate buffered saline pH 7.4 (PBS) for 72 h with four buffer changes in order to remove the salts and stored at -20°C for further use.

#### GC-MS Analysis

The chemical profiling of PPCS was carried out by gas chromatography-mass spectrometry (GC-MS) analysis on PerkinElmer Clarus 600°C equipped with DB-5MS (30 m × 0.25 mm; 0.25 μm film thickness) combined silica capillary column and flame-ionization detector (FID). The PPCS was diluted with ethyl acetate (10 μL/mL) and 1 μL solution was used for analysis in split-mode of 1:30. Helium was the carrier gas with a flow of 1 mL/min, and temperature for injector and sensor were set at 250°C and 280°C, respectively. The column temperature was linearly programmed from 40 to 280°C at 4°C/min and documentation of mass spectra was recorded in EI mode (70 eV) with a range of m/z 40–450 amu. The Turbo Mass software application was adopted to operate and acquire data from GC-MS. The individual components of PPCS were determined based on the MS spectra of accessible reference libraries (NIST/Wiley). The concentration (%) of constituent components were obtained from GC peak area with devoid of FID response factor.

#### MALDI-TOF Analysis

The Matrix Assisted Laser Desorption/Ionization Time-Of-Flight (MALDI-TOF) spectrum of PPCS dialysate was acquired on an Ultra flex Bruker mass spectrometer (Billerica, MA, USA). An analyte was prepared by mixing equal amounts of PPCS with saturated 0.1% TFA and methanol (1:1), and injected into 100-well stainless steel by dried drop method. The analysis was carried out in a linear positive mode with a nitrogen laser at 337 nm and 15 kV. The analysis was carried in multiple times to obtain clear spectra and measured masses have an error of ∼±3 Da.

### Antioxidant Activity

The DPPH free radical scavenging activity of PPCS was determined following the methodology of Kalagatur et al. (2015) with minor modifications. A volume of 1 mL of acetate buffer, 1 mL of ethanol and 0.5 mL of 0.25 mM DPPH solution were added to the different volumes of PPCS and incubated under dark at room temperature for 30 min. The solution mixture without test sample and with quercetin was referred as control and standard, respectively. Following the incubation period, the absorbance was recorded at 517 nm using multimode plate reader (Bioteck Synergy H1, USA). The result was stated as IC_50_ (μL/mL), which represents the quantity of PPCS required to reduce the absorbance of DPPH by 50%. The DPPH free radical scavenging activity of PPCS was calculated by the formula,

DPPH⁢ radial⁢ scavenging⁢ activity(%)=[(Ac−At)/Ac]×100

Where, Ac was the absorbance of the control and At was the absorbance of the test sample (PPCS or standard).

### Antifungal Activity

*Fusarium graminearum* (MTCC 1893) positive for ZEA biosynthesis was obtained from the Microbial Type Culture Collection Center and Gene Bank (MTCC), Chandigarh, India, and grown on CD agar for 7 days at 28°C and spores were recovered using peptone water containing 0.001% Tween 80 with a soft scrape. The spore number was estimated using haemocytometer and spore number was adjusted to 1 × 10^6^ per mL. The antifungal activity of *P. pentosaceus* was determined by dual culture overlay technique in terms of zone of inhibition. The antifungal activity of PPCS was determined by micro-well dilution and scanning electronic microscopic methods and also the mechanism of antifungal activity was determined by estimating ergosterol and ROS.

#### Dual Culture Overlay Technique

Antifungal activity of *P. pentosaceus* was determined by dual culture overlay technique following the methodology of Magnusson and Schnürer (2001) with minor modifications. A two cm line of *P. pentosaceus* was streaked on MRS agar plates and incubated for 48 h at 28°C under aerobic condition. Following the incubation period, the plates were overlaid with MES agar and inoculated with 10 μL fungal spore suspension (1 × 10^6^ spores/mL) and further incubated for 7 days at 28°C. The zone of growth inhibition was measured in mm using Zonescale (HiMedia, Mumbai, India).

#### Micro-Well Dilution

The antifungal activity of PPCS was determined in terms of minimum inhibitory concentration (MIC) by micro-well dilution technique in 96 well microtiter plates following the methodology of CLSI and Kalagatur et al. (2015) with minor modifications. A volume of 10 μL fungal spore suspension (1 × 10^6^ spores/mL) was added to the different volumes of PPCS and final volume was adjusted to 200 μL with CD broth. The wells with 0.001% Tween 80 and 10 μL spore suspension (1 × 10^6^ spores/mL) were referred as control and neomycin were used as a standard. The plate was incubated for 3 days at 28°C and optical density was recorded at 620 nm using multimode plate reader (Synergy H1, BioTek, USA). The minimum concentration at which absolutely inhibited fungal growth was determined as MIC value. The inhibition of fungal growth was calculated by the formula,

Growth⁢ inhibition⁢ (%)=At/Ac×100

Where, Ac and At are absorbance of control and test sample (PPCS or standard), respectively.

#### Scanning Electron Microscopic Observation

The antifungal activity of PPCS on the mycelial structure of *F. graminearum* was determined by SEM observation following the methodology of Kalagatur et al. (2015) with minor modifications. A 3 days old culture of *F. graminearum* grown on CD agar was treated with an IC_50_ value of PPCS and incubated at 28°C for 24 h. Subsequently, the mycelia were washed for twice with PBS and attached to dual adhesive carbon tape and subjected to drying under CO_2_. The mycelia were sputter coated with gold and the morphological feature was observed under SEM (FEI, USA) at 20.0 KV in environmental mode. The mycelia without PPCS treated was referred as a control.

#### Determination of Ergosterol

The determination of ergosterol content in *F. graminearum* was carried out following the methodology of Silva et al. (2010) with slight modifications. A volume of 10 μL spore suspension (1 × 10^6^ spores/mL) and different volumes of PPCS were added to 100 mL of CD broth in Erlenmeyer flask and incubated at 28°C at 160 rpm for a time period of 7 days. The flask alone with fungal spore suspension in CD broth was referred as a control. Following the incubation period, a 50 mg of mycelia was blended with 5 mL of methanol, 1 mL of ethanol and 0.5 g of potassium hydroxide under 250 rpm for 20 min and incubated for 40 min in water bath at 70°C. The supernatant was recovered by centrifugation (8000 rpm for 10 min) and blended with an equal volume of n-hexane for 2 min at 8000 rpm and incubated for 10 min at room temperature. The mixture was completely dried over a water bath at 60°C and dissolved in 1 mL of methanol. The optical density was measured at excitation and emission wavelength of 240 nm and 300 nm, respectively, using UV-3600 spectrophotometer (Shimadzhu, Japan). The quantification of ergosterol content was carried out in Nexera UHPLC (Shimadzu, Kyoto, Japan) equipped with the C18 column, 5 μm, 250 mm × 4.60 mm (Phenomenex, USA). The analysis was carried with following conditions: mobile phase was acetonitrile and water (6.5:3.5, v/v), injection volume was 25 μL and UV detector was used with excitation and emission wavelengths of 240 nm and 300 nm, respectively. The concentration of ergosterol was determined from the standard calibration curve of ergosterol.

#### Determination of ROS

Effect of PPCS on the generation of ROS in *F. graminearum* was carried out in 96 well microtiter plates following the methodology of Kalagatur et al. (2016). A volume of 10 μL fungal spore suspension (1 × 10^6^ spores/mL) was added to the different volumes of PPCS and final volume was adjusted to 200 μL with CD broth, incubated at 28°C for 3 days. The wells with 0.001% Tween 80, 10 μL spore suspension (1 × 10^6^ spores/mL) and neomycin were referred as control and standard, respectively. After incubation, 5 μM of DCFH-DA was added to wells and incubated for 20 min in the dark and absorbance was measured at excitation and emission wavelength of 495 nm and 550 nm, respectively, in a multimode plate reader (Synergy H1, BioTek, USA). ROS was determined as percentage release with respect to the carrier (0.001% Tween 80) treated control. The images were captured using fluorescence microscope EVOS (Life Technologies, USA) at an excitation wavelength of 495 nm and an emission wavelength of 550 nm.

### Antimycotoxic Activity of PPCS

A volume of 10 μL fungal spore suspension (1 × 10^6^ spores/mL) and different volumes of PPCS (20, 40, and 66 μL/mL) were added to the 100 mL of CD broth in 250 mL Erlenmeyer flask. The flask with fungal spore suspension and 100 mL of CD broth was considered as control. The flasks were incubated at 28°C for 14 days and broth culture was separated out from mycelia using Whatman no.1 filter paper. The ZEA concentration in broth culture was determined using UHPLC (Shimadzu, Kyoto, Japan) system equipped with fluorescent detector.

#### Determination of Mycelial Biomass

The mycelia were washed twice with PBS and collected in pre-weighed Whatman no.1 paper and dried for 24 h at 60°C. The dry weighed of mycelia biomass was determined using Denver instruments (Bangalore, India).

#### Determination of ZEA

The determination of ZEA in broth culture was carried out following our previous established methodology (Kalagatur et al., 2015). The selective extraction of ZEA was carried out using ZEA specific immunoaffinity column obtained from Vicam, USA. The column was pre-conditioned with PBS for 10 min and used for ZEA analysis. The broth was blended with an equal amount of acetonitrile for a period of 30 min, and centrifuged for 12 min at 6000 rpm and a volume of 15 mL supernatant was passed through ZEA immunoaffinity column. The column was washed with 5 mL of PBS and repeated with 10 mL distilled water and dried out. The ZEA was eluted with 5 mL of acetonitrile by maintaining contact between column antibodies for 5 min. The eluant was dried over a water bath at 60°C and extract was redissolved in 1 mL of acetonitrile and filtered through 0.22 μm filter, and used for the quantification of ZEA. The quantification of ZEA was carried out in Nexera UHPLC (Shimadzu, Kyoto, Japan) equipped with the C18 column, 5 μm, 250 mm × 4.60 mm (Phenomenex, USA). The analysis was carried out in reverse phase with following conditions: mobile phase was acetonitrile and water (6:4, v/v) with flow rate of 1 mL/min, injection volume was 25 μL and fluorescence detector was used with excitation and emission wavelengths of 334 nm and 450 nm, respectively. A calibration curve for standard ZEA (100 ng – 5 μg/mL) was constructed and used for determination of ZEA in test samples.

#### Reverse Transcriptase qPCR (Rt-qPCR) Analysis of ZEA Metabolic Pathway Genes

The *PKS4* and *PKS13* genes are involved in ZEA biosynthesis in *F. graminearum* (Kim et al., 2005; Gaffoor and Trail, 2006). The effect of PPCS on gene expression of *PKS4* and *PKS13* were analyzed by Rt-qPCR method. The fresh mycelia were flash-frozen with liquid nitrogen and ground into fine powder, and total RNA was extracted using RNA easy plant mini kit following the guidelines of the manufacturer (Qiagen, USA). The total RNA was quantified using NanoDrop 8000 Spectrophotometer (Thermo Scientific, USA) and stored at -20°C. The primers used for the target genes (*PKS4, PKS13*, and *GAPDH*) amplification (**Table 1**) were followed as per our previous report (Kalagatur et al., 2015). The Rt-qPCR analysis was carried in the Light cycler 480 (Roche, USA) using iScript One-Step RT-PCR Kit with SYBR Green master mix (BIO-RAD, Bangalore, India). In brief, 50 μL volume of reaction mixture consists of 25 μL of 2X SYBR Green master mix, 1 μL of iScript reverse transcriptase, 1 μL of primer (450 nM), 1 μL of template RNA (100 ng) and 22 μL of nuclease free water (PCR grade). The reaction conditions were as follows: 10 min of cDNA synthesis at 50°C for 1cycle, 5 min of polymerase activation at 95°C and followed by 35 cycles of PCR at 95°C for 10 s, 60°C for 30 s for combined annealing and extension. The relative quantitative expression of each gene was obtained from a second derivative maximum analysis by determination of the crossing point value normalized to the crossing points values of reference gene *GAPDH*. Data were shown as normalized ratios of genes together with a standard error by means of Roche Applied Science E-Method (Tellmann and Geulen, 2006).

### Statistical Analysis

In the present study, all the experiments were carried out independently in six times and data were processed by one-way ANOVA following the Tukey’s test. The result was considered significant at *p* < 0.05 and represented as means ± SD.

## Result and Discussion

### Molecular Identification of LAB

Isolation of LAB was carried out using selective agar medium (MRS) and pure colonies were isolated by streak plated technique and stored at 4°C for further use. To know the molecular identity of the isolated LAB culture 16S rRNA sequencing was carried out and sequence was submitted to NCBI. BLAST analysis of 16S rRNA revealed that, the isolated LAB was *P. pentosaceus* and obtained sequence was identified with NCBI accession number: SUB1500777 *Pediococcus* KX214298.

### Preparation and Profiling of PPCS

*Pediococcus pentosaceus* cell-free supernatants were prepared and concentrated by ammonium sulfate based precipitation method as reported elsewhere. The chemical profile of PPCS was determined by GC-MS and MALDI-TOF analysis.

#### GC-MS Analysis

Chemical profile of PPCS was carried out by GC-MS analysis and results of the present study revealed that, there was a presence of several antioxidants including phenylpropanoic acid, 3-phenyllactic acid, 3-hydroxydecanoic acid, hydroferulic acid, *trans*-caffeic acid, catechol, benzeneacetic acid, 4-hydroxybenzoic acid, 3-(3-hydroxyphenyl) propionic acid, and cyclic dipeptides including D and L- pro (**Table 2**). These results are in line with the earlier report of Mozzi et al. (2010). In the present study identified phenolic compounds in PPCS could be responsible for the determined antifungal activity. Also, these results are strongly supported by the reports of Dalie et al. (2010), for the antagonistic activity of PPCS against *F. proliferatum* and *F. verticillioides.* The antifungal activity of PPCS in the present study may be due to the presence of phenyllactic acid, 2-hydroxy-4-methylpentanoic acid, reuterin, benzoic acid, and lactic acid (Lavermicocca et al., 2003; Ndagano et al., 2011).

**Table 2:** Chemical characterization of *Pediococcus pentosaceus* culture supernatant (PPCS) by GC-MS analysis.

S. No	Retention time	Compound	Composition (%)
1	10.26	D-Pro	3.05
2	10.47	L-Pro	2.91
3	12.09	Benzeneacetic acid	12.46
4	12.84	Catechol	1.79
5	14.91	Phenylpropanoic acid	6.90
6	17.58	Salicylic acid	8.14
7	19.90	Palmitic acid	3.16
8	20.94	3-Phenyllactic acid	21.08
9	21.88	4-Hydroxybenzoic acid	4.91
10	23.49	Oleic acid	2.60
11	24.05	3-(3-Hydroxyphenyl) propionic acid	1.15
12	25.81	3-Hydroxydecanoic acid	5.71
13	27.01	Hydroferulic acid	3.59
14	34.08	*trans*-Caffeic acid	4.27

		Total	81.72

#### MALDI-TOF Analysis

**FIGURE 1:**
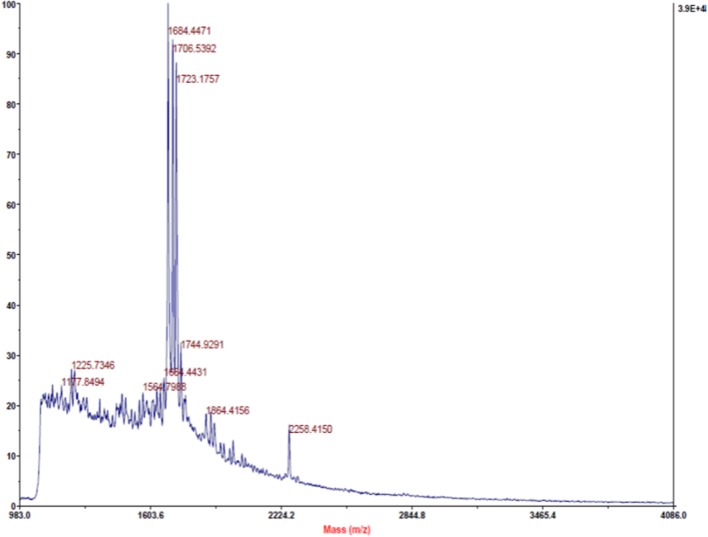
**Matrix-Assisted Laser Desorption/Ionization Time-Of-Flight (MALDI-TOF) identification of peptides from *Pediococcus pentosaceus* culture supernatant (PPCS)**.

In the present study, MALDI-TOF analysis of PPCS was carried out to know the presence of antimicrobial peptides. Study results revealed that there was the presence of three major peptides with a molecular mass of 1684.44, 1706.53, and 1723.17 (**Figure 1**). These results are in line with the earlier reports of (Singh et al., 2014), who reported the presence of a non-pediocin like peptide with a potent antimicrobial activity. In addition to this, we also identified other two uncharacterized peptides and presence of these peptides in PPCS might be the possible cause of determined antifungal activity in the present study. However, structural and functional characterization of these peptides is needed in detail to understand the mechanism of antifungal activity and role of PPCS.

### Antioxidant Activity

**FIGURE 2:**
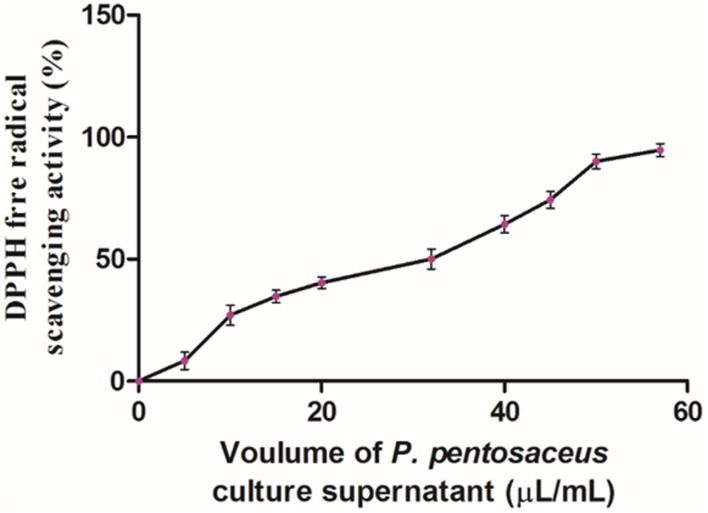
**Antioxidant activity of PPCS determined by DPPH free radical scavenging assay.** Data were processed using one-way ANOVA following Tukey’s test and *p* value was significant (<0.05).

DPPH assay is one of the reliable and sensitive methods for determination of antioxidant potential. The DPPH free radical gets scavenged by hydrogen donating electron group of antioxidant and the value was measured in IC_50_, which means the amount of antioxidant required to scavenge 50% of DPPH free radicals (Bondet et al., 1997). In the Present study, antioxidant potential of PPCS was carried out by DPPH free radical scavenging assay. Results of the present study showed that, the antioxidant potential of PPCS was dose-dependent in a manner (**Figure 2**) and the IC_50_ value was determined as 32 ± 1.89 μL/mL. In the present study, obtained result was in accordance with the previous reports of Balakrishnan and Agrawal (2014) who reported the enhancement of antioxidant potential (%) of cow milk, goat milk and camel milk fermented with *P. pentosaceus* as 38.7 ± 1.53 to 78.0 ± 0.74, 54.0 ± 1.02 to 92.7 ± 1.53, and 64.4 ± 01.42 to 84.3 ± 2.09, respectively. The determined antioxidant activity may due to the presence of phenolic compounds and other secondary metabolites (Okcu et al., 2016) or antimicrobial peptides (Nishino et al., 2000).

### Antifungal Activity

**FIGURE 3:**
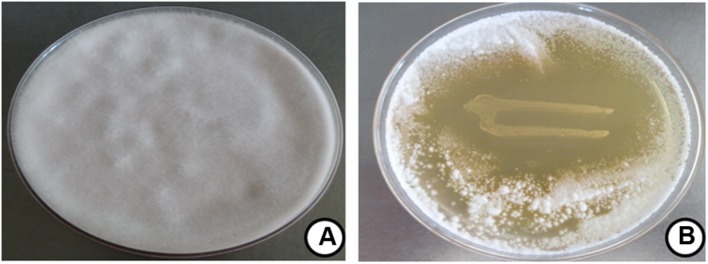
**Antifungal activity of *P. pentosaceus* on *F. graminearum* determined by dual culture overlay technique.** Data were processed using one-way ANOVA following Tukey’s test and *p* value was significant (<0.05). Fungal growth at **(A)** control and **(B)** *P. pentosaceus* streaked plate.

The antifungal property of *P. pentosaceus* cells was determined by dual culture overlay technique and zone of inhibition was observed as 47 ± 2.81% (**Figure 3**). The observed results were in line previous report of Magnusson et al. (2003), who observed the potent antifungal activity of *P. pentosaceus, L. acidophilus* and *L. plantarum* against *A. fumigatus* J9, *A. nidulans J283* (FSGC A4 wt), *F. sporotrichioides* J304 (ITEM168), *P. commune* J238 (IBT 12400), and *P. roqueforti* J268 (IBT 6754). In another study, Laitila et al. (2002) reported that, the antifungal activity of *L. plantarum* E76 and *L. plantarum* E98 on *F. graminearum* as 49 ± 8 and 50 ± 5, respectively. However, till date there is no such reports are available on antifungal activity of *P. pentosaceus* on *F. graminearum.*

**FIGURE 4:**
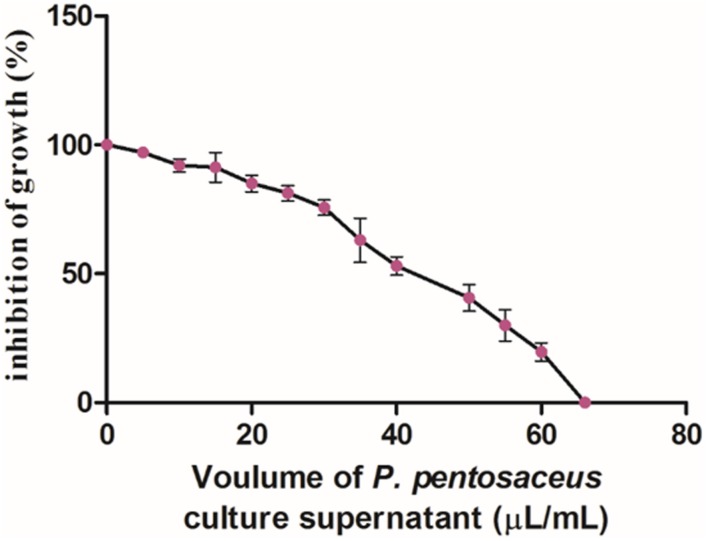
**Dose-dependet antifungal activity of PPCS determined by micro-well dilution method.** Data were processed using one-way ANOVA following Tukey’s test and *p* value was significant (<0.05).

In the present study, the antifungal activity of PPCS was determined by micro-well dilution method and results clearly revealed that there was dose-dependent decrease in growth of *F. graminearum* (**Figure 4**). The MIC value of PPCS against *F. graminearum* was determined as 66 ± 2.18 μL/mL in broth culture and these results are in line with the antifungal activity of *P. pentosaceus* studied by dual culture overlay technique. Moreover, results of the present study was in accordance with the previous report of Ndagano et al. (2011), who reported the antifungal activity of LAB culture supernatants on *A. niger*CWBIF194, *A. niger*MUCL 28699, *A. tubingensis* MP1, and *P. crustosum*MY1.

**FIGURE 5:**
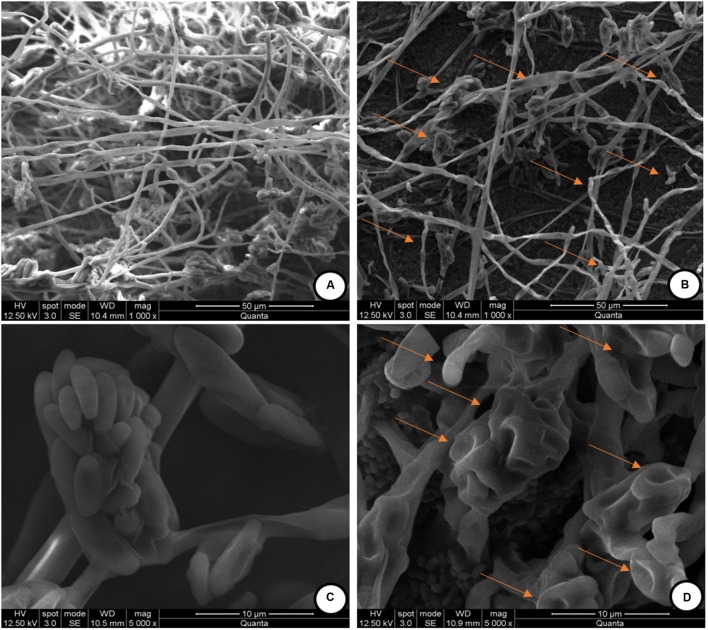
**Scanning electron microscopic (SEM) observation of mycelia of *F. graminearum* exposed with *P. pentosaceus* culture supernatant (PPCS). (A)** Control mycelia, **(C)** control spores and, **(B,D)** were mycelia and spores, respectively treated with minimum inhibitory concentration (MIC) of *P. pentosaceus* culture supernatant (PPCS).

The observed antifungal activity of PPCS was further validated by using SEM analysis to check the mycelial micro-morphological alterations in PPCS treated *F. graminearum.* The mycelia treated with determined MIC value of PPCS was used for SEM analysis. The SEM observation of *F. graminearum* was clearly evidenced that, control mycelia were showed healthy morphology with smooth, turgid, homogenous surface without any apparent change (**Figure 5A**). On the other hand, the morphology of PPCS treated mycelia were observed with notable changes in mycelia including shrinkage, wrinkle, disturbance, collapse, squash, non-homologous surface, and vesicles (**Figure 5B**). Moreover, the conidia treated with PPCS (**Figure 5D**) also exhibited irregular surface, shrinkage, craters and protuberance in structure compared to control (**Figure 5C**). These results are in line with the earlier reports of Zhao et al. (2014) and Kalagatur et al. (2015) who studied the morphological effect of *B. subtilis* SG6 and *O. sanctum* L. essential oil on mycelia structure of *F. graminearum*.

**FIGURE 6:**
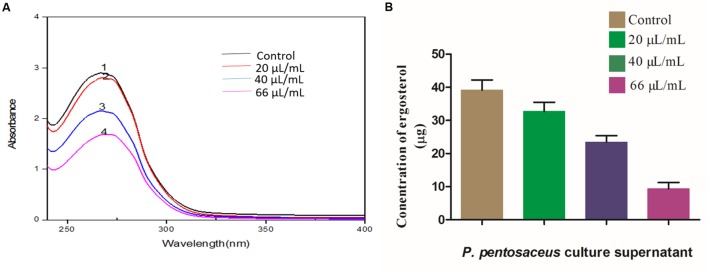
**Dose-dependent effects of *P. pentosaceus* culture supernatant (PPCS) on ergosterol biosynthesis of *F. graminearum* determined by (A) UV- spectrophotometry and (B) UHPLC analysis.** Data were processed using one-way ANOVA following Tukey’s test and *p* value was significant (<0.05).

Ergosterol is a potent bio-marker for determination of fungal growth (Feng et al., 2005), and in the present study, we determined the effect of PPCS on ergosterol content of *F. graminearum*. Results evidenced that, PPCS effectively decreased the content of ergosterol in a dose-dependent manner as determined by UV- spectrophotometer (**Figure 6A**). The concentration of ergosterol was quantified by UHPLC, the levels of ergosterol in control was observed as 39.16 ± 2.91 μg, whereas in PPCS treated test samples observed with dose-dependent decrease in the concentration of ergosterol, such as 32.71 ± 2.94 μg in 20 μL/mL of PPCS, 23.48 ± 1.03 μg in 40 μL/mL of PPCS and the lowest concentration of 9.34 ± 1.86 μg was recorded at a concentration of 66 μL/mL of PPCS treatment (**Figure 6B**). Interestingly these results are in agreement with the mechanistic effect of synthetic chemical antifungal agents currently being used for control of fungi, including amphoteric B, miconazole, itraconazole, and clotrimazole. Mode of action of these chemical fungicides, as it binds and creates pores in the membrane, and inhibits the synthesis of ergosterol and in turn leads to the osmotic imbalance, oxidative apoptotic stress and membrane damage (Groll and Kolve, 2004). Therefore, the obtained low ergosterol concentrations in the PPCS treated samples in the present study strongly support the antifungal activity of PPCS through mitigation of ergosterol metabolism.

**FIGURE 7:**
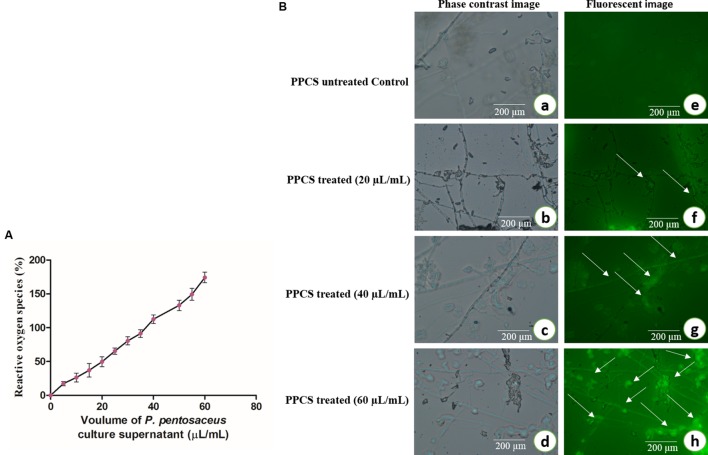
**Antifungal activity of PPCS on *F. graminearum*. (A)** Effect of PPCS on the generation of reactive oxygen species (ROS). **(B)** Phase contrast (a–d) and fluorescent images (e–h) of PPCS untreated and treated mycelia. Data were processed using one-way ANOVA following Tukey’s test and *p* value was significant (<0.05).

Also in another study of ROS, results showed that generation of ROS was increased on increasing concentration of PPCS treatment (**Figure 7A**). In a biological system, ROS plays an important role in cell signaling and homeostasis, and under stress conditions levels of ROS increases and leads to the damage in the cell membrane, oxidative stress and finally brings apoptosis (Kalagatur et al., 2016). The levels of ROS were observed under a fluorescent microscope and images were observed with higher intensity of fluorescence on increasing concentration of PPCS compared to untreated control (**Figure 7B**) and this could be also responsible for the antifungal activity of PPCS (Kalagatur et al., 2016).

Also, analysis of ROS clearly suggests that there is the discrete formation of free radicals in PPCS treated mycelium compare with the untreated control (**Figure 7**). The ROS is the main mechanism involved in oxidative stress thus lead to cause the apoptotic cell death. Recently Kalagatur et al. (2016) also reported the role ROS in the apoptotic mycelial death of *F. graminearum.*

### Antimycotoxic Activity of PPCS

**FIGURE 8:**
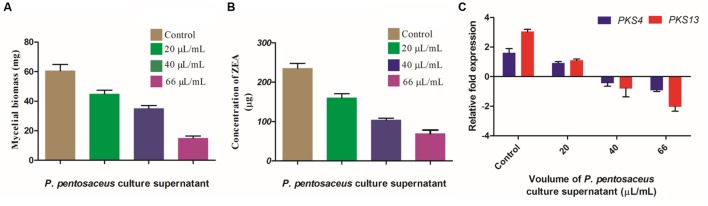
**Effect of PPCS on (A) mycelial biomass, (B) zearalenone (ZEA) synthesis, and (C) expression levels of *PKS4* and *PKS13* (ZEA metabolic genes).** Data were processed using one-way ANOVA following Tukey’s test and *p* value was significant (<0.05).

The main aim of the present study was to evaluate the inhibitory activity of PPCS on ZEA production of *F. graminearum* in broth culture. The effect of PPCS was determined on fungal mycelial biomass, expression of ZEA biosynthetic pathway genes (*PKS4* and *PKS13*) and production of ZEA (**Figure 8**). Results of the present study revealed that the mycelia biomass at 20, 40, and 66 μL/mL of PPCS treated *F. graminearum* broth cultures were determined as 44.84 ± 2.17, 35.02 ± 1.66, and 14.93 ± 1.12 mg, respectively. Whereas, 60.58 ± 3.74 mg of mycelia biomass was observed in PPCS untreated control and the mycelial biomass was decreased with increasing the concentration of PPCS (**Figure 8A**). The concentration of ZEA was quantified by UHPLC analysis and results showed that, ZEA concentration was inversely proportional to the PPCS concentration (**Figure 8B**) and the concentration of ZEA was observed as 235.10 ± 6.92 μg in PPCS untreated control, whereas 160.19 ± 5.80 μg in 20 μL/mL, 103.65 ± 2.05 μg in 40 μL/mL, and 69.12 ± 5.14 μg in 66 μL/mL were recorded in PPCS treated test samples (**Figure 8B**).

To check the effects of PPCS on ZEA metabolic pathway gene expression and decreased ZEA production in broth culture, we analyzed the target ZEA metabolic pathway genes expression by Rt-qPCR analysis. Results clearly evidenced that, there is a dose-dependent down-regulation of target gene expression when compared to the untreated controls. The down-regulated expression levels of *PKS4* and *PKS13* were quantified as 0.70 ± 0.08 and 1.93 ± 0.02 in 20 μL/mL, 2.05 ± 0.14 and 3.81 ± 1.09 in 40 μL/mL, 2.5 ± 0.06 and 5.04 ± 0.80 in 66 μL/mL of PPCS treated samples, respectively, compared to control (1.60 ± 0.82 for *PKS4* and 3.03 ± 0.10 for *PKS13*) and showed in **Figure 8C**. Ponts (2015) reported that stress responses including abiotic (pH, temperature, light, aeration *etc*.) and biotic (surrounding living organisms) factors cause oxidative burst and as a result fungi release certain secondary chemical metabolites as defense agents called mycotoxins. In our previous study, we demonstrated that antioxidants of *O. sanctum* essential oil were effective in controlling the ZEA synthesis through down-regulating the ZEA metabolic pathway genes (*PKS4* and *PKS13*) by Rt-qPCR analysis. In the present study, the observed high antioxidant activity of PPCS was responsible for the decrease in ZEA production through down-regulating *PKS4* and *PKS13* genes.

## Conclusion

*Fusarium graminearum* cause a severe problem in agriculture and food industry. There is a need for proficient and safe ways to control the growth and mycotoxins of *F. graminearum*. Our results evidently indicated that, the antifungal potential of *P. pentosaceus* and its culture supernatant. The PPCS showed the potential inhibitory activity on ZEA production by decreasing the mycelial biomass and down-regulating the ZEA metabolic pathway genes (*PKS4* and *PKS13*). This could be elucidated by the effect of antifungal compounds on the structure and function of nuclear DNA through oxidative nuclear apoptosis mechanisms. Also in the present study, it was observed that, there was a clear lineage among mycelial biomass, target gene expression as well as ZEA production in broth cultures. However, to know the exact mechanism by which PPCS inhibiting the ZEA production by *F. graminearum* in broth cultures yet to be known.

## Author Contributions

All the authors are equally contributed in the contributed in the present work, including planning and assisted in carrying entire lab work, statistical analysis, and final draft making.

## Conflict of Interest Statement

The authors declare that the research was conducted in the absence of any commercial or financial relationships that could be construed as a potential conflict of interest.
